# Maternal and umbilical cord blood lactate for predicting perinatal death: a secondary analysis of data from a randomized controlled trial

**DOI:** 10.1186/s12887-023-04008-y

**Published:** 2023-04-18

**Authors:** Milton W. Musaba, Brendah Nambozo, David Mukunya, Julius Wandabwa, Justus K. Barageine, Paul Kiondo, Agnes Napyo, Quraish Sserwanja, Andrew D Weeks, James K Tumwine, Grace Ndeezi

**Affiliations:** 1 Department of Obstetrics and Gynaecology, Busitema University/ Mbale Regional Referral and Teaching Hospital, Mbale, Uganda; 2 Sanyu Africa Research Institute, Mbale, Uganda; 3 Department of Public and Community Health, Busitema University Faculty of Health Sciences, Mbale, Uganda; 4 Department of Obstetrics & Gynaecology, School of Medicine, Makerere University College of Health Sciences, Kampala, Uganda; 5 Sanyu Research Unit, University of Liverpool, University of Liverpool/Liverpool Women’s Hospital, Liverpool, UK; 6 Programmes Department, GOAL Global, Khartoum, Sudan; 7 Department of Paediatrics and Child Health, School of Medicine, Makerere University College of Health Sciences, Kampala, Uganda

**Keywords:** Lactate, Perinatal-mortality, Obstructed labour, Stillbirth, Neonatal death

## Abstract

**Background:**

In high resource settings, lactate and pH levels measured from fetal scalp and umbilical cord blood are widely used as predictors of perinatal mortality. However, the same is not true in low resource settings, where much of perinatal mortality occurs. The scalability of this practice has been hindered by difficulty in collecting fetal scalp and umbilical blood sample. Little is known about the use of alternatives such as maternal blood, which is easier and safer to obtain. Therefore, we aimed to compare maternal and umbilical cord blood lactate levels for predicting perinatal deaths.

**Methods:**

This was secondary analysis of data from a randomized controlled trial assessing the effect of sodium bicarbonate on maternal and perinatal outcomes among women with obstructed labour at Mbale regional referral hospital in Eastern Uganda. Lactate concentration in maternal capillary, myometrial, umbilical venous and arterial blood was measured at the bedside using a lactate Pro 2 device (Akray, Japan Shiga) upon diagnosis of obstructed labour. We constructed Receiver Operating Characteristic curves to compare the predictive ability of maternal and umbilical cord lactate and the optimal cutoffs calculated basing on the maximal Youden and Liu indices.

**Results:**

Perinatal mortality risk was: 102.2 deaths per 1,000 live births: 95% CI (78.1–130.6). The areas under the ROC curves were 0.86 for umbilical arterial lactate, 0.71 for umbilical venous lactate, and 0.65 for myometrial lactate, 0.59 for maternal lactate baseline, and 0.65 at1hr after administration of bicarbonate. The optimal cutoffs for predicting perinatal death were 15 0.85 mmol/L for umbilical arterial lactate, 10.15mmol/L for umbilical venous lactate, 8.75mmol/L for myometrial lactate, and 3.95mmol/L for maternal lactate at recruitment and 7.35mmol/L after 1 h.

**Conclusion:**

Maternal lactate was a poor predictor of perinatal death, but umbilical artery lactate has a high predictive value. There is need for future studies on the utility of amniotic fluid in predicting intrapartum perinatal deaths.

## Introduction

In Uganda, neonatal mortality is 19 deaths per 1000 live births [1] while the still birth risk is 17 deaths per 1000 live births [2]. Intrapartum birth asphyxia accounts for 47% of these perinatal deaths [3]. Fetal scalp and umbilical cord lactate are widely used in high resource settings to predict perinatal outcome [4, 5]. However, it is not always feasible to obtain fetal scalp blood during birth as it requires expertise. Furthermore, obtaining the sample at the time of birth might be a little too late to enable commencement of life saving interventions such as intrauterine resuscitation [6] and preparations for helping an asphyxiated newborn to initiate breathing within the golden minute. On the other hand, fetal scalp sampling is not very popular because of the risk of infection spread, especially in low resource settings such as sub-Saharan Africa where HIV/AIDS and other sexually transmitted diseases are prevalent [7, 8].

Therefore, the prospect of predicting neonatal outcomes in the intrapartum period using a maternal sample is exciting. Maternal samples would make it easier to measure lactate at the bedside and institute timely interventions such as neonatal resuscitation and specialist consultations if required. Studies from both high and low resource settings have reported the association between fetal scalp and umbilical blood lactate with perinatal mortality [9, 10]. However, there is a dearth of data on the predictive ability of maternal blood lactate. Furthermore, there is no information on ideal cut-offs for maternal lactate to predict short term perinatal outcomes.

In this study, we hypothesized that maternal lactate is a good predictor of perinatal mortality and aimed to compare maternal and umbilical blood lactate as predictors of perinatal death.

## Materials and methods

### Study design

We conducted a prospective cohort study, where women with obstructed labour were consecutively recruited into the clinical trial at time of diagnosis. This was a double blind, randomized controlled trial. Preoperatively, half of the patients received an infusion of sodium bicarbonate (intervention arm) and the other half received no sodium bicarbonate (control arm) [11] and the mother-baby pairs were followed up to the 7th day postpartum.

### Study setting

This is a secondary analysis of data from a study performed in the labour ward of Mbale regional referral hospital in Eastern Uganda [11] between July 2018 and September 2019. The hospital serves a population of about four million people from 16 districts and one city. Mbale hospital is the main referral center for four district hospitals and ten health sub-districts in and around Mount Elgon zone. Annually, 12,000 deliveries are conducted and about 600 of these are diagnosed with obstructed labour [12].

### Study participants

Participants were mother-baby pairs that participated in the randomized controlled trial to establish the effect of sodium bicarbonate on maternal and perinatal outcomes among women with obstructed labour in Mbale regional referral hospital.

### Inclusion criteria

We included patients with obstructed labour carrying singleton term pregnancies (≥ 37 weeks of gestation) in cephalic presentation, with a live baby at enrolment [11].

### Exclusion criteria

We excluded patients without a fetal heart at the time of enrollment, patients with other obstetric emergencies such as antepartum haemorrhage, pre-eclampsia and eclampsia (defined as elevated blood pressure of at least 140/90 mm Hg, urine protein of at least 2+, any of the danger signs and seizures), premature rupture of membranes and intrauterine foetal death. Patients with comorbidities such as diabetes mellitus, sickle cell disease, renal disease, liver disease and heart disease.

### Study procedure

All women diagnosed with obstructed labour by an obstetrician or medical officer using a definition of the American Association of Obstetricians and Gynecologists (ACOG) [13] were screened and all eligible participants enrolled into the trial (Registration PACTR20180500334421) [11]. Midwives in the delivery suite were sensitized about the ongoing project and informed the study team of all potential participants. Two research assistants were available throughout the day and night with the aim of recruiting eligible women [11]. All participants received the standard preoperative care recommended by the Ministry of Health. This includes antibiotic prophylaxis, intravenous fluids (1.5 L of normal saline), bladder drainage and lying in the left lateral position in preparation for emergency caesarean section.

### Lactate measurement

We measured lactate levels in both umbilical and maternal venous blood at the bedside using a hand-held Lactate Pro2 device (Arkray, Japan Shiga). Maternal blood lactate was measured at enrollment, at one hour after administration of study drug, and from the myometrium at cesarean section (this was the case in 90% of the participants). The myometrial sample was collected using a 2 ml syringe from the initial incision on the lower segment of the uterus before entry into the endometrial cavity. At birth, two samples were obtained from a doubly clamped segment of the umbilical cord.

### Power and sample size

We had 548 women diagnosed with obstructed labour and screened for inclusion into the randomized controlled study. All the mother - baby pairs were included in this analysis [14].

## Main variables

### Outcome variable

The outcome variable of interest was perinatal death. This was defined as the demise of a fetus from the time the mother was recruited into the trial up to the 7th postnatal day. We included both fresh still births and early neonatal deaths.

### Exposure variables

Data was collected on several intrapartum factors from recruitment to discharge. These included: maternal age, maternal height, maternal weight, parity, history of rupture of membranes, history of being referred from a lower health facility, history of using traditional medicines (majorly used for induction, augmentation and analegesia) in labour, birth weight, maternal blood lactate, myometrial blood lactate, and umbilical cord blood lactate. The maternal pulse, blood pressure and fetal heart rate were collected at time of recruitment/measurement of baseline lactate. Maternal and umbilical cord blood lactate levels were our main exposure variables.

### Data collection

A team of six trained research assistants who were also qualified midwives collected data. An electronic interviewer administered questionnaire aided the open data kit software on password protected android tablets from enrollment up to time of discharge or 7th day postnatal as detailed in the trial protocol [11]. The research assistants were trained on how to measure lactate using the Lactate Pro2 device (Arkray, Japan Shiga) point of care device under the supervision of the principal investigator (PI) or his designee. We saved the collected data on a password protected aggregate server to which only the PI had access, for conducting daily checks to ensure completeness of the uploaded questionnaires.

### Data analysis

Data were analyzed using Stata version 17.0 (Stata Corp LLC, College Station, Texas, United States of America). We summarized continuous variables using means with standard deviations or medians with interquartile ranges and categorical variables using their frequencies and percentages.

We constructed receiver operator curves (ROC) to compare the predictive ability of maternal lactate at baseline and one hour after administration of study drug, myometrial lactate, umbilical venous and arterial lactate at birth using Stata’s ‘roccomp’ command. Optimal cut-off values for predicting perinatal mortality and early neonatal mortality were determined for maternal lactate on admission with obstructed labour and one hour after administration of study drug, myometrial lactate, and umbilical venous and arterial lactate at birth using the maximal Youden Index and Liu’s index. Youden’s index objectively calculates the optimal cut-off. It takes into account the sensitivity and specificity and it is used to estimate the diagnostic effectiveness of various cut off points [15] whereas Liu’s index calculates: the optimal cut off point, while maximizing the product of sensitivity and specificity [16].

### Ethics

#### Ethical approval

to conduct the study was obtained from the Makerere University School of Medicine Research and Ethics Committee (#REC REF 2017 − 103) and the Uganda National Council for Science and Technology (HS217ES). Administrative clearance was obtained from the Mbale regional referral hospital research and Ethics Committee (MRRH-REC IN-COM 00/2018). Written informed consent was obtained from each of the participants before enrolment.

## Results

**Fig. 1:**
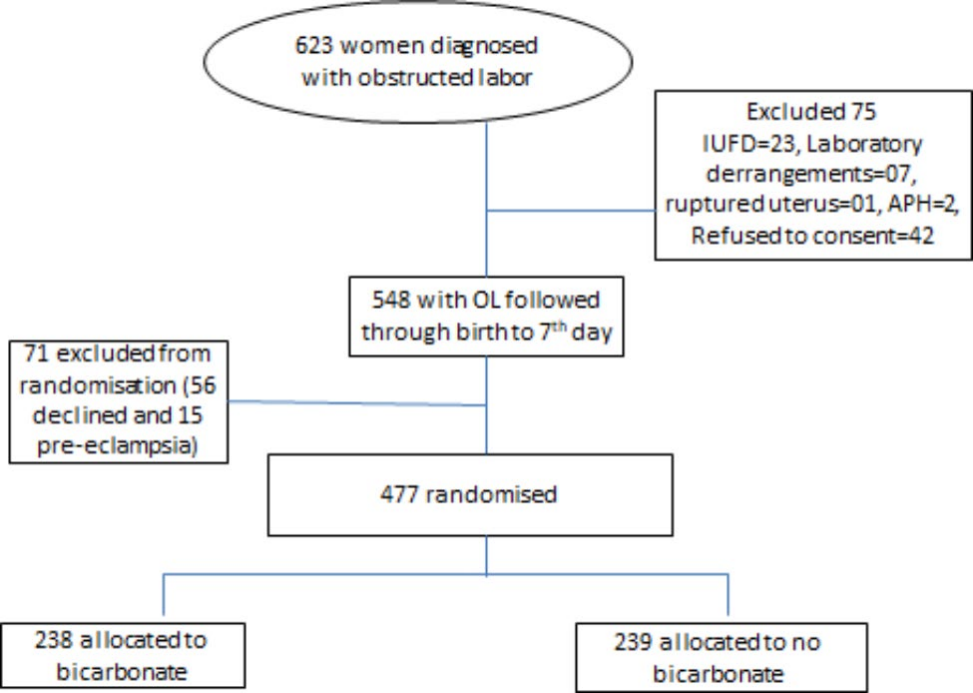
Flow chart of recruitment of women with obstructed labour in Eastern Uganda

A total of 623 women were diagnosed with obstructed labour, 75 were excluded while 548 women and their babies met the inclusion criteria for final analysis. The details are in Fig. 1 [11].

### Participant characteristics

The median maternal age was 23 years (IQR 19–27) while the median gestational age was 38 weeks (IQR 38–40). Majority 545 (99.45%) of the participants had attended antenatal care while slightly more than half 295 (53.8%) were primi gravid. Two thirds, 348(63.5%) of the participants were referred from district hospitals and health centers, and 317(57.8%) had used local herbs while in labour. Most women 446(81.39%) had premature rupture of membranes. The rest of the results are shown in Table 1.

### Perinatal mortality

Fresh still birth risk was 43.8 deaths per 1,000 live births: 95% CI (28.3–64.4). Early neonatal mortality risk was: 58.4 deaths per 1,000 live births: 95% CI (40.3–81.4). Perinatal mortality risk was: 102.2 deaths per 1,000 live births: 95% CI (78.1–130.6).

**Fig. 2:**
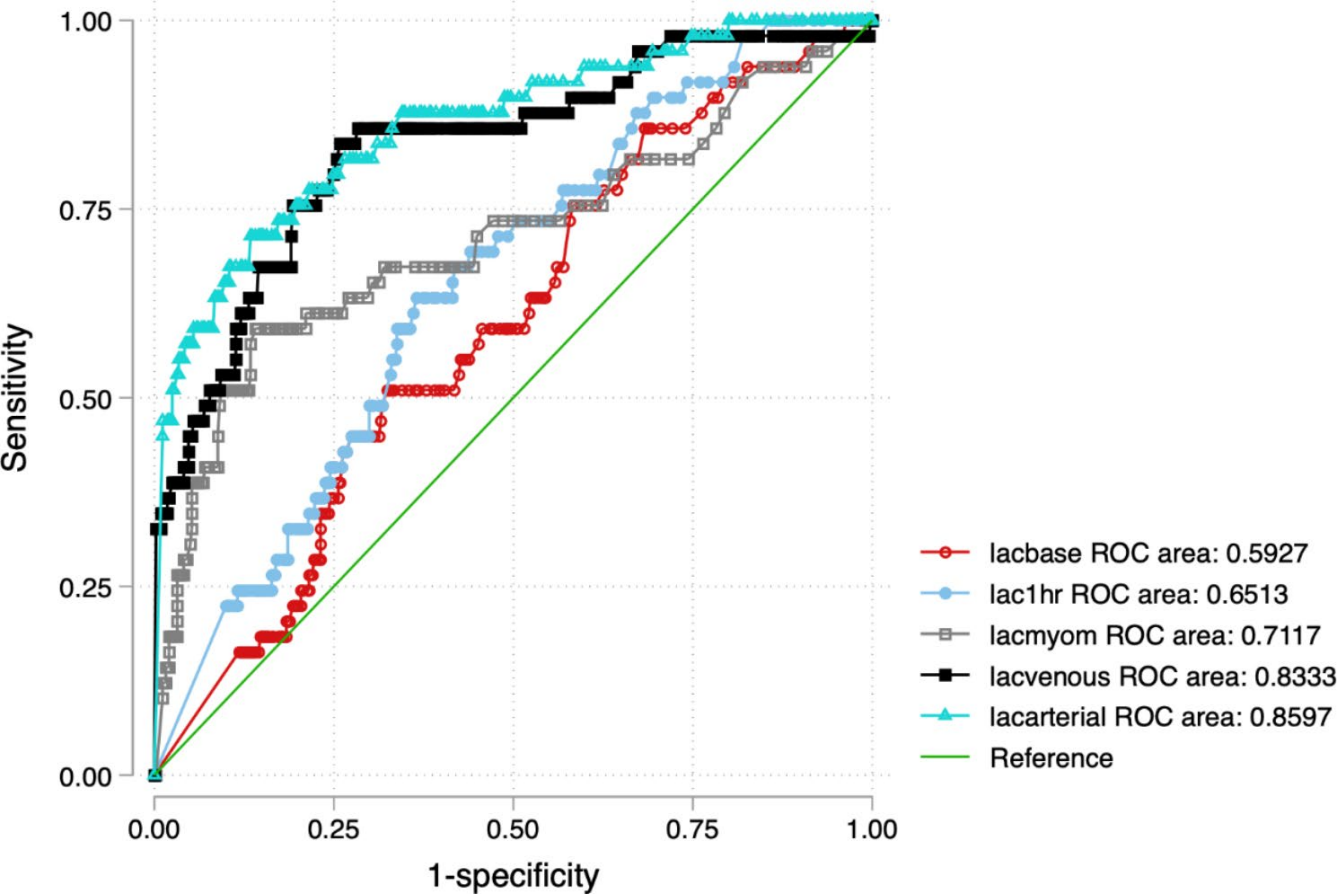
Comparison of receiver operating characteristic curve areas of maternal and umbilical lactate values in predicting perinatal mortality

The areas under the curve were 0.86 for umbilical arterial lactate, 0.83 for umbilical venous lactate, 0.71 for myometrial lactate, 0.65 for 1 h lactate, and 0.59 for baseline lactate 0.59 for predicting perinatal mortality. Details are in Fig. 2; Table 2.

**Fig. 3:**
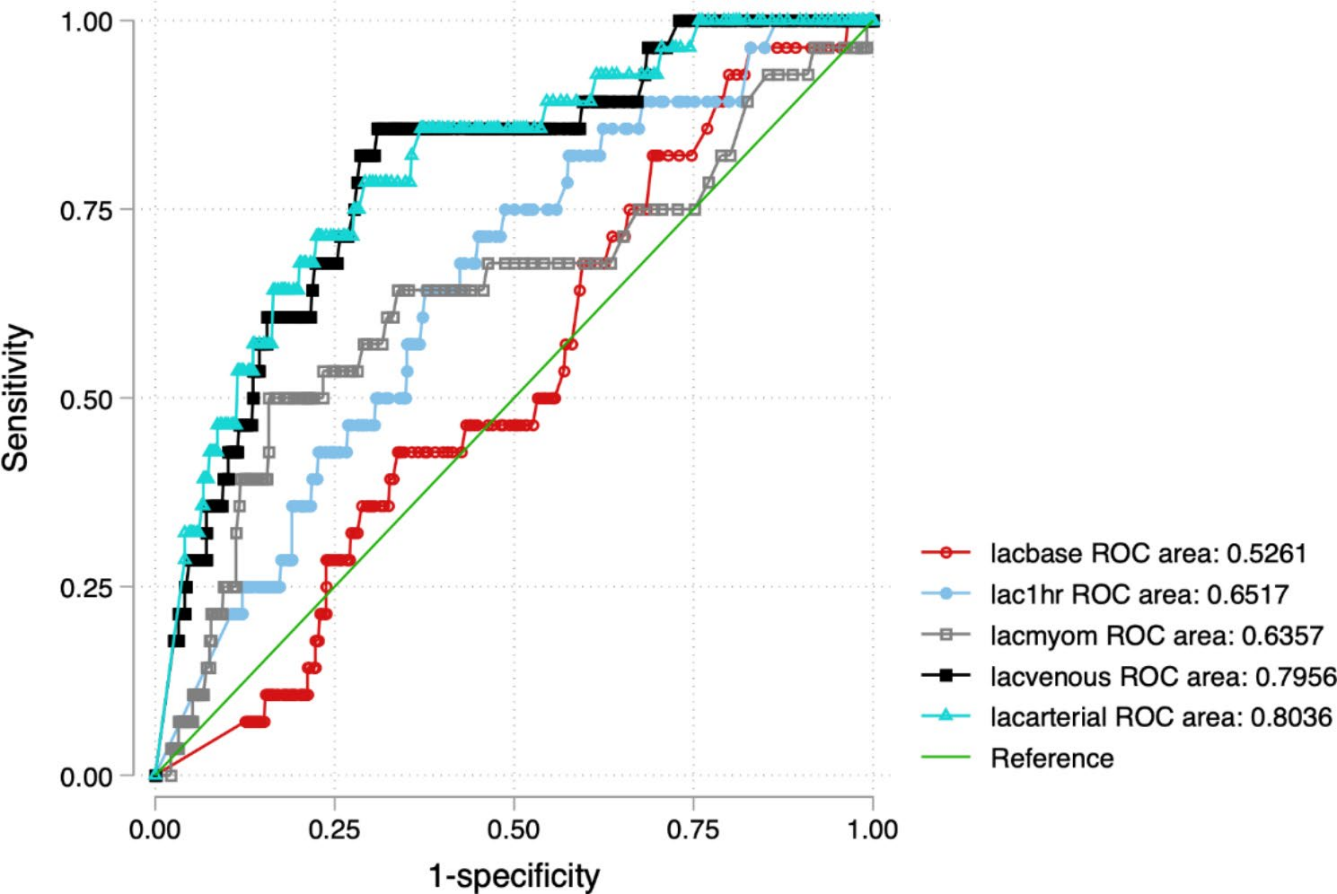
Comparison of receiver operating characteristic curve areas of maternal and umbilical lactate values in predicting early neonatal mortality

The areas under the curve were 0.80 for umbilical arterial, 0.79 for umbilical venous, 0.63 for myometrial, and 0.65 1 h lactate, 0.52 baseline lactate for predicting early neonatal deaths. The details are in Fig. 3; Table 2.

### Optimal cut offs for predicting perinatal death

Based on the maximal Youden and Liu indices, the optimal cutoffs for predicting perinatal death were 15.85mmol/L for umbilical arterial lactate, 11.35mmol/L for umbilical venous lactate, 8.65mmol/L for myometrial lactate, 4.85 mmol/L for maternal lactate. The optimal cutoffs for predicting early neonatal death were 10.75mmol/L for umbilical arterial lactate, 10.15mmol/L for umbilical venous lactate, 8.75mmol/L for myometrial lactate, 3.95 mmol/L for maternal lactate. Details are in Table 3.

**Table 1:** Characteristics of women with obstructed labour in Mbale Hospital

Variables	Alive (n = 492)	Dead (n = 56)	Total (n = 548)
**Birth weight, Kg**			
Normal (2.5–3.5)	335 (68.1%)	34 (60.7%)	369 (67.3%)
Low (< 2.5)	14 (2.9%)	2 (3.6%)	16 (2.9%)
High (> 3.5)	143 (29.1%)	20 (35.7%)	163 (29.7%)
**Use of herbal medicine in labour**			
No	218 (44.3%)	13 (23.2%)	231 (42.2%)
Yes	274 (55.7%)	43 (76.8%)	317 (57.9%)
**Referral status**			
No	192 (39.0%)	8 (14.3%)	200 (36.5%)
Yes	300 (61.0%)	48 (85.7%)	348 (63.5%)
**Gravidity**			
2 to 4	162 (32.9%)	17 (30.4%)	179 (32.7%)
PG	266 (54.1%)	29 (51.8%)	295 (53.8%)
5+	64 (13.1%)	10 (17.9%)	74 (13.5%)
**Maternal age, years**			
20 to 35	334 (67.9%)	31 (55.4%)	365 (66.6%)
<20	133 (27.0%)	21 (37.5%)	154 (28.1%)
>35	25 (5.1%)	4 (7.1%)	29 (5.3%)
**History of membrane**			
No	97 (19.7%)	5 (8.9%)	102 (18.6%)
Yes	395 (80.3%)	51 (91.1%)	446 (81.4%)
**Attended ANC**			
**Yes**	489 (99.4%)	56 (100.0%)	545 (99.5%)
**No**	3 (0.6%)	0 (0.0%)	3 (0.6%)
*Gestation age	38 (38–39)	38.5 (38–40)	38 (38–40)
*Mother’s weight	64 (58–71)	61 (56–69.5)	63 (58–70)
*Mother’s height	159 (154–164)	156.5 (151–163)	159 (154–164)
*Mother’s Pulse rate	88 (78–98)	87.5 (76.5–108)	88 (78–99)
*SBP	123 (113–132)	119.5 (109.5–130)	123 (113–132)
*DBP	78 (70–83)	71 (65–80)	78 (69–83)
*FHR	136 (130–142)	134 (100–148)	136 (130–143)
*Birth weight of newborn	3.3 (3–3.6)	3.4 (2.9–3.6)	3.3 (3–3.6)
*Days in hospital	3 (2–4)	3 (3–4)	3 (2–4)

*For continuous variables, I present median and IQR

**Table 2:** Comparison of receiver operating characteristic curve areas of maternal and lactate values in predicting perinatal and early neonatal mortality

	Maternal Lactate baseline	Maternal Lactate 1 h	Myometrial Lactate	Umbilical Venous Lactate	Umbilical Arterial lactate	P value
	ROC area (95% CI)	ROC area (95% CI)	ROC area (95% CI)	ROC area (95% CI)	ROC area (95% CI)	
Perinatal mortality	59.3% (51.3–67.2)	65.1% (57.6–72.6)	71.2% (61.9–80.4)	83.3% (76.6–90.1)	86.0% (79.8–92.1)	< 0.0001
Neonatal Mortality	52.6% (42.8–62.4)	65.2% (55.4–74.9)	63.6% (51.2–75.9)	79.6% (71.3–87.8)	80.4% (72.1–88.7)	0.0003

**Table 3:** Comparison of cut off values for predicting perinatal death

	Maternal Lactate baseline		Maternal Lactate 1 h		Myometrial Lactate		Umbilical Venous Lactate		Umbilical Arterial lactate	
	Index		Index		Index		Index		Index	
	Youden	Liu	Youden	Liu	Youden	Liu	Youden	Liu	Youden	Liu
**‘Optimal’ cut-off for perinatal mortality**	4.85	4.85	7.65	8.8	8.65	8.65	11.35	11.35	15.85	14.45
Sensitivity at cut-off	79%	79%	70%	64%	59%	59%	80%	80%	66%	70%
Specificity at cut-off	42%	42%	57%	62%	86%	86%	75%	75%	87%	83%
AUC at cut-off	60%	60%	63%	63%	73%	73%	78%	78%	77%	76%
**‘Optimal’ cut-off for early neonatal mortality (1–7 days)**	3.95	4.85	7.35	7.75	8.75	5.85	10.15	10.85	10.75	10.75
Sensitivity at cut-off	84%	72%	75%	72%	50%	64%	84%	81%	81%	81%
Specificity at cut-off	31%	40%	53%	56%	84%	66%	67%	69%	63%	63%
AUC at cut-off	58%	56%	64%	64%	67%	65%	76%	75%	72%	72%

## Discussion

In this study, maternal blood lactate was found to be a poor predictor of both perinatal mortality at seven days postnatal. On the other hand, umbilical cord lactate was a good predictor of perinatal mortality at seven days postnatal. This finding is not surprising because the process of labour is characterized by intermittent periods of hypoxia of variable duration, characterized by anaerobic breakdown of glucose in the myometrial cells [17]. This inefficient process of generating ATP molecules produces a lot of lactate as a by-product, within the fetal placental unit. Lactate levels are reported to be highest in amniotic fluid compared to maternal blood [18] and subsequently much of the by products such as lactate arise from the feto-placental unit [19]. This finding is further supported by our observation that of the maternal blood samples, myometrial lactate measured at cesarean section was a better predictor of perinatal death, probably because it was nearer the source of lactate in fetal placenta. Therefore, it is not surprising that high umbilical cord blood lactate is a specific marker for metabolic acidosis that is associated with more neonatal complications [20]. Our findings are similar to those of previous studies, which have showed that umbilical lactate is a good predictor of other perinatal outcomes other than death [21, 22]. However, umbilical blood measurements are obtained too late to alter the timing of intervention by CS. Furthermore, in low-income settings, there are logistical difficulties associated with obtaining umbilical blood samples in the immediate postnatal period when the focus is on immediate neonatal resuscitation and maternal care. Nonetheless, tracking of the umbilical lactate can identify neonates for target therapy and neonatal care to try to ameliorate the effects of hypoxia and ischemia. In addition to the potential for educational, medicolegal and professional benefits associated with this practice [23].

We therefore recommend further studies to explore the possibility of measuring lactate from other samples such as amniotic fluid that can be easily collected during labour.

Our study found a much higher cutoff value (15.5mmol/L) for umbilical arterial lactate compared to other studies whose cutoffs ranged between 3.75 and 8 mmol/L [21–23]. This difference could be attributed to the fact that women with obstructed labour have higher levels of blood lactate compared to those that labour normally [18]. Therefore, there is need for more studies to establish the ideal cut offs of umbilical cord lactate outside a randomised a randomised controlled trial setting. In addition, this may also be due to the fact that different studies used other methods to measure blood lactate levels. For instance point of care devices for measuring lactate are not uniformly calibrated [24, 25]. We used the latest version of the lactate Pro 2 devise, while earlier studies used meters from other manufactures [26]. Our choice of this device was informed by the fact that it did not require any calibration before and during use.

### Methodological considerations

To our knowledge, this is the first report comparing maternal and umbilical cord lactate for predicting perinatal death among women with obstructed labour. Our estimate of perinatal death could have been much higher if it was not in a setting of a clinical trial where every effort was made to ensure that each of the enrolled women was delivered in less than two hours of diagnosis. In addition, half of our sample had been administered sodium bicarbonate, but this had no significant effect on the results as seen in the maternal lactate results at one hour after study drug administration.

## Conclusion

Maternal blood lactate is a poor predictor of perinatal death, compared to umbilical cord blood lactate. We suggest that future studies should explore the use of other maternal samples like amniotic fluid for predicting perinatal mortality.

## Data Availability

The datasets used and/or analysed during the current study are available from the corresponding author on reasonable request.
